# New insights from genetic studies of eczema

**DOI:** 10.1515/medgen-2023-2010

**Published:** 2023-04-05

**Authors:** Ingo Marenholz, Aleix Arnau-Soler, Oscar Daniel Rosillo-Salazar, Young-Ae Lee

**Affiliations:** Max Delbrück Center for Molecular Medicine in the Helmholtz Association (MDC) Robert-Rössle-Str. 10 13125 Berlin Germany; Max Delbrück Center for Molecular Medicine in Robert-Rössle-Str. 10 13125 Berlin Germany; Max Delbrück Center for Molecular Medicine in the Helmholtz Association (MDC) Robert-Rössle-Str. 10 13125 Berlin Germany; Max Delbrück Center for Molecular Medicine in the Helmholtz Association (MDC) Robert-Rössle-Str. 10 13125 Berlin Germany

**Keywords:** Eczema, atopic dermatitis, allergy, filaggrin, epidermal differentiation complex, complex disease, genome-wide association study, rare variants

## Abstract

Genome-wide association studies (GWAS) provided fundamental insight into the genetic determinants of complex allergic diseases. For eczema, 58 susceptibility loci were reported. Protein-changing variants were associated with eczema at genome-wide significance at 12 loci. The majority of risk variants were, however, located in non-coding, regulatory regions of the genome. Prioritized target genes were enriched in pathways of the immune response and of epithelial barrier function. Interestingly, a large overlap in the genetic architecture underlying different allergic diseases was identified pointing to common pathomechanisms for eczema, asthma, hay fever, and food allergy. Here, we review the most recent findings from GWAS for eczema including the role of rare variants and genetic heterogeneity in ethnically diverse populations. In addition, we provide an overview of genes underlying Mendelian disorders featuring eczematous skin inflammation.

## Introduction

Eczema (atopic dermatitis) is a chronic inflammatory skin disease with a lifetime prevalence of up to 20 % in westernized countries. It usually develops in infancy and often precedes or co-occurs with other allergic phenotypes, such as food allergy, asthma, and hay fever. Eczema is characterized by chronic relapsing skin inflammation of flexural areas of the body, severe itch, and an aberrant immune response to environmental allergens. A defective skin barrier is a hallmark of eczema, manifesting clinically as marked dryness, sensitivity to physical/chemical irritation, and increased sensitivity to bacterial and viral infections.

Eczema is a multifactorial, complex disease. The increasing prevalence over the last decades clearly pointed to environmental factors as important modifiers of disease risk. On the other hand, family and twin studies indicated that up to 80 % of eczema susceptibility can be attributed to heritable factors, making the genome the target of interest for deciphering the molecular mechanisms underlying eczema. While loss-of-function (LOF) mutations in the epidermal barrier gene filaggrin (*FLG*) identified 16 years ago are still the strongest genetic risk factors for eczema, genome-wide approaches unraveled a number of involved pathways and contributed a plethora of candidate genes including some promising targets for therapeutic approaches.

In this article, we summarize the latest findings on the genetics of eczema. We review studies investigating the role of common and rare variants and of genetic determinants shared between different allergic diseases. Finally, we discuss the influence of the ethnic background which may contribute to differences in eczema phenotype and prevalence.

## Searching for the causal eczema variants

In order to identify susceptibility genes in complex diseases such as eczema, different genome-wide strategies were applied. Whole genome linkage studies investigated allele sharing among affected relatives. However, their requirement for either extended pedigrees or large numbers of nuclear families or affected sib pairs as well as the lack of precision in mapping complex disease genes limited their feasibility and wide-spread use.

The advent of array technology enabled the genotyping of up to several million single nucleotide polymorphisms (SNPs) per individual in a single experiment and quickly replaced linkage studies with genome-wide association studies (GWAS). GWAS compare the allele frequencies of SNPs in affected cases and unaffected control individuals. Due to the case-control design, study participants are much easier to recruit. Moreover, the high density of markers and the linkage disequilibrium between them enabled the mapping of susceptibility loci to much smaller chromosomal intervals.

## Genome-wide association studies identify common variants—importance of the eczema definition

Since 2009, over 15 GWAS were conducted reporting 58 eczema susceptibility loci (Tables 1 and 2). The majority of studies was performed in populations of European [1–9] and East Asian ancestry [10–15]. Three studies included populations of diverse ancestries (Table 1). To gain statistical power, several meta-analyses were conducted which combined the summary results of individual GWAS in a single study [2, 7–9, 15]. The largest meta-GWAS on eczema comprised 22,474 cases and 774,187 controls from the Estonian Biobank, FinnGen, and the UK Biobank (UKB) [8]. Many of the reported susceptibility loci were found in more than one GWAS (Table 2). It should, however, be noted that due to the meta-analysis design study populations partially overlapped between different studies (Table 1). Hence, replication was not always independent.

Large study populations recruited in nationwide efforts, such as the UKB, FinnGen, Estonian Biobank, or BioBank Japan (BBJ), are of great value for genetic studies, but the quality of the phenotype information on eczema may vary. While in FinnGen, Estonian Biobank, or BBJ codes of the International Statistical Classification of Diseases, 10th Revision (ICD-10) were available for eczema, the UKB provided this information only for a small subset of individuals. In the UKB, two data fields were related to eczema; a questionnaire-based report of a diagnosis of “rhinitis/hay fever or eczema ever” and a self-report of “eczema or dermatitis”. However, both data fields did not capture eczema with the required accuracy; asking adults for “rhinitis/hay fever or eczema” would preferentially identify individuals with allergic rhinitis since it is a much more common disorder. Moreover, the trait “eczema or dermatitis” would include a large number of individuals with unspecified dermatitis. An acceptable solution to this problem was to use the overlap of both definitions which would yield fewer cases but enrich for eczema [9, 16]. Accordingly, the multi-ethnic eczema GWAS, meta-analyzing results from BBJ (n= 2,597 cases) with data on “rhinitis/hay fever or eczema” from UKB (n= 25,685 cases), should be regarded as a study on allergic disease rather than eczema. All susceptibility loci reported as newly associated with eczema in this meta-analysis [14] were found in a previous GWAS on any allergic disease [16] and were therefore excluded from Table 2. Likewise, two GWAS reporting results on eczema but using either the “rhinitis/hay fever or eczema” or the “eczema or dermatitis” definition in the UKB were not included here [17, 18].

## Studying rare variants in eczema—explaining the missing heritability?

In recent years, more and more studies indicated a substantial role of rare variants in complex diseases [19, 20]. Their identification on the genome-wide level is however challenging. Since the penetrance of individual variants in complex diseases is incomplete, large numbers of samples need to be genotyped in order to find statistically significant differences between cases and controls. In addition, most rare variants are not covered by the commercially available genotyping arrays. Therefore whole exome sequencing (WES) or whole genome sequencing (WGS) are the methods of choice for rare variant detection. Since genome-wide sequencing studies are cost-intensive, they were applied to a limited number of severely affected individuals or family members. WES performed in eczema patients with ichthyosis vulgaris from Ethiopia [21], in individuals with eczema and high IgE levels from Japan [22], and in adult-onset eczema patients from Korea [23] identified rare nonsense and missense mutations in a number of epidermal differentiation complex (EDC) genes, in *ADAM33*, *DSG4*, *GTF2H5*, *EVPL*, *NLRP1, SPINK5*, and *CYP27A1*. However, due to limited sample size and power (n < 50 cases), none of the associations were statistically significant. To understand the genetics underlying human traits more completely, there are increasing efforts for sequencing large study populations: The UKB recently released whole exomes of 454,787 UK individuals. The analyses of rare coding variants in “rhinitis/hay fever or eczema” and “eczema or dermatitis”, both identified 2 LOF mutations in *FLG* at genome-wide significance [24]. Apart from larger study populations, the use of a strict definition of a severe phenotype or severely affected families may improve the power of rare variant studies.

Recently, an alternative, imputation-based approach for studying rare variants in eczema was successfully applied. Since genotyping arrays contain a restricted number of SNPs, imputation is used to estimate missing variants not included on the array from known haplotypes of a reference population. With the Haplotype Reference Consortium panel (HRC) comprising almost 65,000 haplotypes of 32,488 sequenced individuals [25], for the first time a reference set of decent size was available to impute rare variants with high accuracy. In a meta-analysis of 21 study populations including 20,016 eczema patients and 380,433 controls, overall 48 independent variants from 38 loci were associated with eczema of which 11 contained low frequency/rare variants [9]. A similar approach using large population-specific reference panels for imputation was used in a meta-analysis of Estonian Biobank, FinnGen, and UKB reporting 30 loci including 4 rare variants [8]. Finally, genotyping 2,000 eczema patients and 15,000 controls with the Immunochip, a custom genotyping array that was specifically designed for studying chronic inflammatory, immune-related diseases and that included 200,000 common and rare variants from 186 candidate loci, identified low frequency variants in the docking protein 2 gene (*DOK2*) [26].

Imputation-based studies require a minimum number of the rare allele in the reference data set; they cannot identify individual mutations involved in the disease. Future WES and WGS studies will close this gap in the detection of disease causing rare variants. In eczema, variants with a minor allele frequency below 5 % were estimated to account for 23 % of the SNP-based heritability (77 % was explained by common variants), supporting a substantial role for rare and low-frequency variants in eczema risk [9].

## Functional assessment of eczema-associated variants

The majority of variants associated with eczema in GWAS are located in intergenic regions for which a functional link to eczema is often difficult to obtain. Pinpointing the culprit variant(s) at a risk locus, identifying their target gene(s) and experimentally elucidating their role in the disease process remain major challenges. By integrating functional evidence from transcriptomic, proteomic and epigenomic data, including systematic disease-disease and disease-molecular trait colocalization results across 92 cell types and tissues, Mountjoy et al. have recently finemapped >133,000 GWAS loci and made the prioritized target genes publically available [27].

To identify variants with a potential functional impact we screened the GWAS catalog for eczema-associated variants altering the protein sequence or having a high deleteriousness score (Table 3). Apart from the well-established *FLG* LOF mutations, missense variants were reported at genome-wide significance in *RUNX3*, *SLC9A4*, *IL6R*, *IL13*, *DUSP1*, *NOTCH4*, *DOK2,* *NLRP10*, *TESPA*, *NFILZ*, and *TNFRSF6B*. Non-coding variants with a high deleteriousness score were located in *KIAA0391*, *TNXB*, *EGR2*, and *AFF1* and between *PBX2*/*GPSM3*, *CCD80*/*RP11-572C15.3*, and *ZNF365/ADO*. Interestingly, 14 out of 23 variants likely to be harmful had a minor allele frequency < 5 % (Table 3) supporting a role for low frequency and rare variants in eczema.

In eczema, we and others have found a significant enrichment of immune-related pathways involved in cytokine signaling, T-cell differentiation and activation as well as enrichment of genes related to the cornified cell envelope in keratinocytes. Interestingly, stratifying the eczema associated variants according to allele frequency revealed that rare/low-frequency SNPs were more involved in epidermal barrier function whereas common SNPs pointed to immune functions [9].

## Eczema genes from monogenic diseases

A number of eczema-associated genes were identified through rare monogenic diseases, in which eczematous skin inflammation occurs as a symptom of a more complex syndrome. The most prominent discovery was the identification of LOF mutations in the epidermal barrier gene filaggrin (*FLG*). They were first identified as causal variants in families with ichthyosis vulgaris [28], an autosomal-dominant skin disease characterized by dry, scaly skin and often accompanied by eczema. Shortly after, the same mutations were reported to be associated with eczema [29]. Additional eczema risk genes were previously reported for monogenic diseases, including *SPINK5* underlying autosomal-dominant Netherton syndrome, *CARD11* for autosomal-dominant immunodeficiency 11B, and *STAT3* for autosomal-dominant hyper-IgE recurrent infection syndrome. A systematic search for “eczema” or “atopic dermatitis” in OMIM yielded 18 genes mutated in Mendelian disorders commonly presenting with eczema-like skin lesions. Recurrent additional features were immunodeficiency, inflammation, other allergic disorders, and elevated total IgE as well as cutaneous and skeletal abnormalities (Table 4). Some of the underlying genes, such as *FLG*, *IL6R*, *CARD11*, and *IL2RA*, were also reported in GWAS on eczema, others were located in or near eczema susceptibility loci (Table 2). Future studies may identify additional overlaps in the genetics of eczema and rare monogenic diseases.

## Filaggrin—are there more eczema risk genes in the epidermal differentiation complex?

In eczema, LOF mutations in *FLG* show semi-dominant inheritance with incomplete penetrance. With an odds ratio of 3 they are the strongest known risk factor for eczema. Almost all GWAS replicated the *FLG* locus within the EDC [30], a 1.6-Mb region on chromosome 1q21.3 that contains over 60 genes, most of which are expressed during differentiation of the skin or of mucosal tissues. GWAS usually detect multiple common variants significantly associated with eczema in that region and it is tempting to assume that additional susceptibility genes for eczema reside within the EDC. However, a recent GWAS with data on low frequency and rare variants was able to condition the results on the 3 main LOF variants in Europeans; all significant association signals within the EDC were eliminated [9] indicating that they were due to *FLG* mutations. The effect of *FLG* mutations on eczema might be even larger. A systematic evaluation of all *FLG* LOF mutations reported in the gnomAD sequence database, which includes the sequences of 141,456 individuals from populations world-wide [31], revealed that the 4 most common European mutations covered 80 % of the LOF alleles in that population [32]. A total of 276 different mutations accounted for the remaining 20 % of LOF alleles, which could not be imputed due to their very low allele frequencies. In non-European populations, an additional 320 LOF variants in *FLG*, most of them rare, were reported. Thus, sequencing in populations of diverse ancestries will be required to uncover additional rare eczema risk variants not only in the EDC.

## Susceptibility loci shared between eczema and other allergic diseases

Eczema patients often suffer from additional allergic diseases, such as asthma, hay fever, or food allergy, pointing to a common genetic background underlying different allergic disorders. To identify shared risk variants, GWAS on any allergic disease were performed enabling large increases in sample size [16, 33–35]. The largest meta-GWAS on allergic disease compared 180,129 cases with asthma and/or hay fever and/or eczema with 180,709 non-allergic controls from 13 study population of European ancestry [16]. A total of 99 genomic loci with 136 independently associated genetic variants were identified. Among them, only 5 genomic loci showed evidence for a disease-specific effect in patients with only eczema, only asthma, or only hay fever. Interestingly at all 5 loci, allele frequencies of the risk alleles were significantly higher (*FLG*, *IL2RA*) or lower (*L1RL2*/*IL18R1*, *WDR36*/*CAMK4*, *GSDMB*) in eczema, distinguishing this allergic disorder of the skin from the allergic airways diseases asthma and hay fever. On the other hand, eczema and food allergy which often co-occur in infancy seem to share the majority of susceptibility loci. All variants identified in a GWAS on childhood food allergy [36] were located in loci which were also associated with eczema.

Summarizing the results, the majority of eczema loci were also involved in other allergic diseases. The eczema definition as well as the inclusion or exclusion of other allergic traits are important factors influencing GWAS results. Eczema-specific loci exist and the corresponding genes might be involved in epidermal barrier-specific functions.

## The role of the ethnic background—similarities and differences

Heterogeneity of eczema between ethnicities has been described [37]. In East Asian populations, eczema is characterized by immune-dysregulation and epidermal barrier features resembling a phenotype between eczema and psoriasis in patients of European ancestry. African Americans seem to have a greater lesional infiltration of dendritic cells and decreased expression of Th1- and Th17-related markers compared with European Americans. In addition, a higher prevalence of eczema and a less prominent role of *FLG* LOF mutations were observed. GWAS pointed to similarities of the genetic architecture underlying eczema in different ethnic groups but also identified differences which may explain phenotypic heterogeneity.

Of the 58 loci reported here (Table 2), 35 were detected only in studies of European ancestry, 8 were specific for East Asian populations, and 15 were reported in both ethnicities or only in multi-ethnic studies. The excess of European-specific loci may however be attributed to the increased power of these studies due to much larger sample sizes of the respective GWAS; the maximum number of cases was 22,474 in European studies vs. 4,296 in East Asian studies. Apart from ancestry-specific susceptibility loci, genetic diversity is evident on the variant level. Since allele frequencies as well as haplotype structure can vary between populations, different lead SNPs identified at a susceptibility locus in Europeans and Asians, respectively, may be in linkage disequilibrium with the same causal variant. Moreover, also the causal variants may be population-specific. In this regard, a comprehensive analysis of all 600 *FLG* LOF mutations present in the gnomAD database [31] yielded valuable insights [32]. While the mutation spectrum was similar among related populations (e. g. Finnish and non-Finnish European), there was a significant difference between more distant populations. The most common LOF variants in the East Asian population were almost absent in Europeans and vice versa. Interestingly, there was no overlap of the most common LOF mutations between the South Asian population and the East Asian population. The allele frequency of all *FLG* mutations combined ranged from 1.4 % in the Latino/Admixed American to 7.6 % in the East Asian population [32] which indicated an ancestry-dependent contribution of *FLG* LOF mutations in the development of eczema.

To date, genetic research on eczema has mainly been conducted in populations of European and East Asian ancestry. Since ethnic heterogeneity in terms of the eczema phenotype and the underlying genotypes exists, lack of studies on ethnically diverse populations, such as African, South Asian, and Latino populations, is a major drawback as emphasized recently in an article on polygenic risk scores [38]. Future efforts therefore need to be more inclusive regarding ethnicities. This will increase our global knowledge of eczema genetics and may provide benefit to a large proportion of the world population which is not adequately represented in research yet.

## Conclusion

In recent years, GWAS significantly contributed to the identification of genetic loci involved in the development of eczema and shed light on a remarkable overlap of the genetic architecture underlying different allergic diseases. However, the functional impact of the majority of eczema-associated regulatory variants still needs to be resolved. Integration of growing sets of tissue-specific or single-cell-specific transcriptomic, proteomic, and epigenomic data will facilitate the elucidation of underlying disease mechanisms. Moreover, genome-wide sequencing studies will yield new insights into more rare and potentially functional variants.

## Figures and Tables

**Table 1:** Study chracteristics of the genome-wide association studies on eczema.

Studies**^1^**	Year	PUBMED ID	Ancestry	Discovery sample size^2^	Replication sample size	Main studies included	Comment
Esparza-Gordillo et al.	2009	19349984	European	939 /975	1,097 (270 families)		Partially overlapping^3^
Sun et al.	2011	21666691	East Asian (Han Chinese)	1,012 /1,362	3,624 /12,197		
Paternoster et al.	2011	22197932	European	5,606 /20,565	5,419 /19,833		Partially overlapping^3^
Hirota et al.	2012	23042114	East Asian (Japanese)	1,472 /7,971	1,856 /7,021		
Ellinghaus et al.	2013	23727859	European	2,425 /5,449	1,951 /4,599		Partially overlapping^3^, Immunochip^4^
Weidinger et al.	2013	23886662	European	1,563 /4,054	2,286 /3,160		Partially overlapping^3^
Baurecht et al.	2015	25574825	European	2,079 /11,899	NA		Partially overlapping^3^
Kim et al.	2015	25935106	East Asian (Korean)	246 /551	NA		
Schaarschmidt et al.	2015	25865352	European	870 /5,293	1,383 /1,728		Partially overlapping^3^
Paternoster et al.	2015	26482879	European and multi-ethnic	18,900 /84,166 (E) 2,499 /11,298 (NE)	30,588 /226,537 (E) 1,471 /2,091 (NE)	EAGLE consortium,23andme (replication)	
Ishigaki et al.	2020	32514122	East Asian (Japanese)	2,385 /209,651	NA	BBJ	
Tanaka et al.	2021	34116867	East Asian (Japanese) and multi-ethnic^5^	2,597 /110,504 (A) 25,685 /76,768 (E)	NA	BBJ, UKB	Hay fever/eczema in UKB^5^
Sliz et al.	2021	34454985	European	22,474 /774,187	NA	Estonian Biobank, FinnGen, UKB	
Sakaue et al.	2021	34594039	East Asian (Japanese) and multi-ethnic	4,296 /163,807 (A) 6,224 /475,075 (E)	NA	BBJ, FinnGen, UKB	
Grosche et al.	2021	34785669	European	20,016 /380,433	NA	EAGLE consortium, FinnGen, UKB	

BBJ, Biobank Japan; UKB, UK Biobank

^1^ Studies from the GWAS catalog (December 2022) reporting associations with the traits eczema, atopic eczema or atopic dermatitis are shown.

^2^ For multi-ethnic studies, the sample size of each population is indicated; E, European; NE, non-European; A, East Asian.

^3^ The study populations participating in these studies were partially overlapping. The majority of cases was subsequently included in the EAGLE consortium.

^4^ The Immunochip contains 200,000 common and rare variants from 186 candidate loci involved in inflammatory, immune-related diseases.

^5^ In UKB, cases were defined based on the life-time presence of rhinitis/hay fever or eczema. Accordingly, results from the multi-ethnic analysis were excluded from Table 2.

**Table 2:** Genome-wide signifcant loci reported in GWAS on eczema.

Locus number	GWAS locus**^1^**	Chr	Position**^2^**	Lead SNP**^3^**	Ancestry	Study**^4^**	Mapped genes**^5^**	Monogenic disease (gene)
1	1p36.22	1	11988538	rs56243319	M	Sakaue et al.[15]	*MFN2*	
2	1p36.11	1	24964519	rs6672420	E	Grosche et al.[9]	*RUNX3*	
3	1q21.1	1	143595936	rs188069315	E	Sliz et al.[8]	*NBPF17P*	
4	1q21.2	1	147506275	rs1035121917	E	Sliz et al.[8]	*LINC00624*	
5	1q21.2-21.3	1	152033551-152628378	rs78914480, rs187068709, rs558269137, rs61816761, rs3126085, rs189163698, rs12144049, rs12144049, rs6661961, rs61813875, rs72702813	A, A, E, E, A, A, E, E, E, E, E	Ishigaki et al.[13], Sakaue et al.[15], Sliz et al.[8], Grosche et al.[9], Sun et al.[10], Tanaka et al.[14], Baurecht et al.[5], Schaarschmidt et al.[6], Weidinger et al.[4], Paternoster et al.[7], Ellinghaus et al.[3]	*S100A11—LCE3A/LCEP4*	Ichthyosis vulgaris (*FLG*)
6	1q21.3	1	154446273-154454494	rs4576655, rs61812598, rs12126142, rs2228145	M, E, E, E	Sakaue et al.[15], Grosche et al.[9], Sliz et al.[8], Paternoster et al.[7]	*IL6R*	Hyper-IgE recurrent infection syndrome-5 (*IL6R*)
7	1q24.3-25.1	1	172859340-173182897	rs17371133, rs6691738	E, E	Sliz et al.[8], Grosche et al.[9]	*SLC25A38P1/AIMP1P2—GOT2P2/TNFSF4*	
8	2p25.1	2	8302417-8354967	rs891058, rs10199605	E, E	Grosche et al.[9], Paternoster et al.[7]	*LINC00299*	
9	2p13.3	2	70840723-70872975	rs6723629, rs200543403, rs112111458	A, A, E	Sakaue et al.[15], Tanaka et al.[14], Paternoster et al.[7]	*CD207—LINC01143*	
10	2q12.1	2	102350323-102477754	rs1861246, rs13015714, rs3755274, rs6419573, rs368124191, rs5833015, rs5833015, rs759382	E, A, E, E, A, A, A, E	Sliz E, Hirota et al.[11], Grosche et al.[9], Paternoster et al.[7], Tanaka et al.[14], Ishigaki et al.[13], Sakaue et al.[15], Ellinghaus et al.[3]	*IL1RL1/IL18R1—SLC9A4*	
11	2q13	2	111543096	rs1666723	A	Sakaue et al.[15]	*SOCAR*	
12	2q24.3	2	167135776	rs6720763	E	Schaarschmidt et al.[6]	*XIRP2-AS1/XIRP2*	
13	2q37.1	2	233205597	rs62192898	A	Sakaue et al.[15]	*INPP5D*	
14	2q37.3	2	241759225	rs34290285	E	Grosche et al.[9]	*D2HGDH*	
15	3p26.1	3	4984323	rs11130215	M	Sakaue et al.[15]	*BHLHE40*	
16	3p24.3	3	18632606	rs4395418	E	Grosche et al.[9]	*SATB1-AS1*	
17	3p22.3	3	33023847-33045708	rs7613051, rs79497729, rs79497729, rs6780220	A, A, A, A	Hirota et al.[11], Tanaka et al.[14], Ishigaki et al.[13], Sakaue et al.[15]	*GLB1*	
18	3q13.2	3	112657461-112929141	rs12634229, rs75024669, rs72943976, rs12630906, rs9868053	A, A, A, A, E	Hirota et al.[11], Ishigaki et al.[13], Tanaka et al.[14], Sakaue et al.[15], Grosche et al.[9]	*CCDC80/LINC02042—CD200R1*	
19	3q24	3	144401986	rs150979174	E	Sliz et al.[8]	*RNA5SP144—LARP7P4*	
20	4q21.3	4	86939362	rs80199341, rs80199341	A, A	Tanaka et al.[14], Sakaue et al.[15]	*AFF1*	
21	4q27	4	122111486-122576542	rs7666843, rs1904522, rs17389644	E, E, E	Sliz et al.[8], Grosche et al.[9], Ellinghaus et al.[3]	*RN7SL335P/KIAA1109—IL21*	
22	5p13.2	5	35850047-35883632	rs34463936, rs10214237	E, E	Paternoster et al.[7], Grosche et al.[9]	*SPEF2/IL7R—CAPSL*	
23	5q22.1	5	110523120-110818610	rs7701890, rs3853750	A, E	Sun et al.[10], Grosche et al.[9]	*SLC25A46/BCLAF1P1*	
24	5q31.1	5	132655393-132713335	rs12188917, rs1295686, rs1295686, rs1295686, rs1295685, rs848, rs847, rs847, rs11747814, rs2897442	E, E, E, A, A, E, A, E, E, E	Paternoster et al.[7], Weidinger et al.[4], Baurecht et al.[5] Ishigaki et al.[13], Sakaue et al.[15], Ellinghaus et al.[3], Tanaka et al.[14], Sliz et al.[8], Grosche et al.[9], Paternoster et al.[7]	*TH2LCRR—KIF3A*	
25	5q35.1	5	172765347	rs114503346	E	Grosche et al.[9]	*NEURL1B/DUSP1*	
26	6p21.33-21.32	6	32102292-32626262	rs41268896, rs12153855, rs176095, rs3864302, rs11275478, rs4713555, rs13208697, rs28383323	E, E, A, A, A, E, E, E	Baurecht et al.[5], Weidinger et al.[4], Hirota et al.[11], Tanaka et al.[14], Sakaue et al.[15], Paternoster et al.[7], Grosche et al.[9], Sliz et al.[8]	*TNXB—HLA-DRB1/HLA-DQA1*	
27	7p22.2	7	3089155	rs4722404	A	Hirota et al.[11]	*CARD11—AOAH*	Immunodeficiency 11B with atopic dermatitis (*CARD11*)
28	7p21.1	7	20352241-20521373	rs3757723, rs6461503	A	Tanaka et al.[14]	*ITGB8*	
29	8q21.13	8	80363600-80373657	rs11786685, rs10957978, rs6473226, rs6473227	E, M, E, E	Sliz et al.[8], Sakaue et al.[15], Grosche et al.[9], Paternoster et al.[7]	*MIR5708—RNU6-1213P*	
30	8q24.13	8	125597624-125605746	rs6996614, rs12334935	E, E	Sliz et al.[8], Grosche et al.[9]	*TRIB1/LINC00861*	
31	9p21.3	9	22373458	rs10738626	E	Schaarschmidt et al.[6]	*DMRTA1*	
32	9q34.3	9	137605991-137738823	rs117137535, rs3125788	E, A	Grosche et al.[9], Tanaka et al.[14]	*ARRDC1—EHMT1*	
33	10p15.1	10	5996890-6073676	rs6602364, rs62626322	E, E	Paternoster et al.[7], Grosche et al.[9]	*IL15RA/IL2RA—RPL32P23/RBM17*	Immunodeficiency 41 with lymphoproliferation and autoimmunity *(IL2RA*)
34	10q21.2-21.3	10	62593090-62724921	rs4372325, rs10995251, rs10995255, rs10995256, rs438694	E, A, A, A, A	Grosche et al.[9], Hirota et al.[11], Tanaka et al.[14], Ishigaki et al.[13], Sakaue et al.[15]	*ZNF365/ALDH7A1P4*	
35	11p15.4	11	7946812-7960885	rs878860, rs59039403, rs59039403, rs59039403	A, A, A, A	Hirota et al.[11], Tanaka et al.[14], Ishigaki et al.[13], Sakaue et al.[15]	*OR10A3/NLRP10*	
36	11p13-12	11	36343703-36412992	rs10836538, rs12295535, rs11033603	E, E, E	Grosche et al.[9], Ellinghaus et al.[3], Sliz et al.[8]	*PRR5L*	
37	11q13.1	11	65736582-65791795	rs749848972, rs479844, rs479844, rs10791824, rs10791824, rs10791824	A, E, E, M, E, E	Tanaka et al.[14], Paternoster et al.[2], Sliz et al.[8], Sakaue et al.[15], Grosche et al.[9], Paternoster et al.[7]	*KRT8P26/AP5B1—OVOL1*	
38	11q13.5	11	76559639-76621166	rs7130588, rs2212434, rs2212434, rs7110818, rs7936434, rs34455012, rs34455012, rs7927894, rs11236809	E, E, E, E, E, E, A, E, A	Weidinger et al.[4], Baurecht et al.[5], Paternoster et al.[7], Ellinghaus et al.[3], Grosche et al.[9], Sliz et al.[8], Sakaue et al.[15], Esparza-Gordillo et al.[1], Tanaka et al.[14]	*EMSY/LINC02757*	
39	11q23.3	11	118875175	rs10790275	E	Grosche et al.[9]	*Y_RNA*	
40	11q24.3	11	128293268-128551691	rs533495047, rs7127307, rs7127307, rs4245080	E, E, E, M	Sliz et al.[8], Grosche et al.[9], Paternoster et al.[7], Sakaue et al.[15]	*LINC02098—ETS1*	
41	12q13.2	12	54963054	rs183884396	E	Sliz et al.[8]	*TESPA1*	
42	12q13.2-13.3	12	56114625	rs4759228	E	Sliz et al.[8]	*PA2G4/RPL41*	
43	12q15	12	68252741-68258319	rs2227491, rs2227472, rs3947727	E, M, E	Grosche et al.[9], Sakaue et al.[15], Sliz et al.[8]	*IL22—MDM1*	
44	13q21.31	13	64989899	rs9540294	A	Kim et al.[12]	*LGMNP1*	
45	14q13.2	14	35103151-35185261	rs2143950, rs2415269, rs12586305	E, E, E	Paternoster et al.[7], Grosche et al.[9], Sliz et al.[8]	*PPP2R3C/FAM177A1—PRORP*	
46	14q23.3	14	64510603	rs11625265	E	Grosche et al.[9]	*ZBTB1*	
47	14q32.32	14	102790540	rs12888955	E	Grosche et al.[9]	*TRAF3*	
48	16p13.13	16	11100223-11135732	rs3862469, rs2041733, rs2041733, rs2041733	E, E, E, E	Sliz et al.[8], Ellinghaus et al.[3], Grosche et al.[9], Paternoster et al.[7]	*CLEC16A*	
49	17q21.2	17	40608272	rs112401631	E	Grosche et al.[9]	*CCR7/SMARCE1*	Hyper-IgE recurrent infection syndrome 1 (*STAT3*)
50	17q21.31	17	45380520	rs9895436	E	Grosche et al.[9]	*RNA5SP443/ARHGAP27*	
51	17q21.32	17	47795683	rs72833417	E	Grosche et al.[9]	*TBX21/OSBPL7*	
52	17q21.33	17	49345148-49377145	rs35073649, rs16948048, rs16948048, rs28406364	E, E, M, E	Sliz et al.[8], Ellinghaus et al.[3], Sakaue et al.[15], Grosche et al.[9]	*ZNF652—PHB*	
53	18q12.1	18	29549105	rs1361355315	E	Sliz et al.[8]	*RNU6-408P/MIR302F*	
54	18q22.1	18	63945923	rs188720898	E	Sliz et al.[8]	*SERPINB10/HMSD*	
55	19p13.2	19	8677438-8681319	rs2918299, rs2164983, rs3040091, rs2918307, rs35358447	E, E, M, E, E	Grosche et al.[9], Paternoster et al.[4], Sakaue et al.[15], Paternoster et al.[7], Sliz et al.[8]	*NFILZ—ACTL9*	
56	20q13.2	20	54171775-54190682	rs2259735, rs2259735, rs73285914, rs16999165	A, A, E, A	Tanaka et al.[14], Sakaue et al.[15], Grosche et al.[9], Hirota et al.[11]	*CYP24A1—PFDN4*	
57	20q13.33	20	63669458-63697389	rs3848669, rs3848669, rs4809219, rs6010620, rs6010620, rs909341, rs909341	E, E, E, A, A, E, E	Grosche et al.[9], Sliz et al.[8], Paternoster et al.[7], Sakaue et al.[15], Sun et al.[10], Baurecht et al.[5], Ellinghaus et al.[3]	*RTEL1-TNFRSF6B*	
58	22q12.3	22	36918248-36922967	rs4821564, rs2075943	E, E	Grosche et al.[9], Sliz et al.[8]	*CSF2RB*	Immunodeficiency 63 with lymphoproliferation and autoimmunity (*IL2RB*)

M, multi-ethnic; E, European; A, East Asian

^1^ GWAS loci were defined based on a distance > 1Mb between neighbouring genome-wide significant SNPs in the GWAS catalog.

^2^ If multiple studies reported SNP association at a locus, the positions of the first and of the last lead SNP are indicated.

^3^ Associations reported in the GWAS catalog (December 2022) for the traits eczema, atopic eczema or atopic dermatitis are shown. For each locus identified in a study, the lead SNP is indicated. Lead SNPs are ordered according to their genomic positions.

^4^ Association results of the multi-ethnic analysis of Tanaka et al. were excluded due to the eczema definition used for UKB.

^5^ Mapped genes according to the GWAS catalog using the physical position of a SNP are reported for the first and last lead SNP at a locus. For intergenic SNPs the neighbouring genes are listed.

**Table 3:** Eczema-associated variants with potential functional impact.

Gene	SNP ID^1^	Chr	Position	Alleles	AF (EUR)^2^	SNP localization/effect	CADD^3^	Reference
*RUNX3*	rs6672420	1	24964519	T>A	0,521	exonic/missense	25,2	Grosche et al.[9]
*FLG*	rs138726443	1	152307547	G>A	0,997	exonic/stop gained	NA	Grosche et al.[9]
*FLG*	rs558269137	1	152312601	C>CACTG	0,986	exonic/frameshift	18,8	Sliz et al.[8], Sakaue et al.[15]
*FLG*	rs61816761	1	152313385	G>A	0,99	exonic/stop gained	NA	Grosche et al.[9]
*IL6R*	rs2228145	1	154454494	A>C	0,64	exonic/missense	16,0	Paternoster et al.[7]
*SLC9A4*	rs61731289	2	102525124	G>A	0,967	exonic/missense	15,2	Grosche et al.[9]
*CCD80/RP11-572C15.3*	rs12637953	3	112676994	C>T	0,991	intergenic	12,2	Sakaue et al.[15]
*AFF1*	rs79243012	4	86939360	T>C	0,994	intronic	21,3	Sakaue et al.[15]
*AFF1*	rs80199341	4	86939362	T>C	0,995	intronic	21,2	Tanaka et al.[14], Sakaue et al.[15]
*IL13*	rs20541	5	132660272	G>A	0,793	exonic/missense	0,02	Sakaue et al.[15]
*DUSP1*	rs34471628	5	172769749	A>G	0,957	exonic/missense	23,2	Grosche et al.[9]
*DUSP1*	rs34013988	5	172770787	C>T	0,957	exonic/missense	25,1	Grosche et al.[9]
*TNXB*	rs12153855	6	32107027	T>C	0,876	intronic	12,5	Weidinger et al.[4]
*PBX2/GPSM3*	rs176095	6	32190542	A>G	0,796	intergenic	22,4	Hirota et al.[11]
*NOTCH4*	rs8192591	6	32218019	C>T	0,962	exonic/missense	15,8	Grosche et al.[9]
*DOK2*	rs34215892	8	21909729	G>A	0,968	exonic/missense	20,3	Mucha et al. [26]
*ZNF365/ADO*	rs16917691	10	62751018	A>C	0,995	intergenic	15,0	Sakaue et al.[15]
*EGR2*	rs61865882	10	62812333	T>C	0,922	3’UTR	18,6	Tanaka et al.[14]
*NLRP10*	rs59039403	11	7960885	G>A	0,999	exonic/missense	23,1	Tanaka et al.[14], Ishigaki et al.[13], Sakaue et al.[15]
*TESPA1*	rs183884396	12	54963054	G>A	0,995	exonic/missense	29,9	Sliz et al.[8]
*KIAA0391*	rs2415269	14	35169731	G>A	0,73	intronic	14,8	Grosche et al.[9]
*NFILZ*	rs2918299	19	8677438	C>T	0,827	exonic/missense	9,3	Grosche et al.[9]
*RTEL-TNFRSF6B*	rs909341	20	63697389	C>G	0,798	exonic/missense	20,5	Baurecht et al.[5], Ellinghaus et al.[3]

SNP, single nucleotide polymorphism; Chr, chromosome; NA, not available

^1^ Genome-wide significant nonsense/missense variants and variants with a high CADD score (>12) from the GWAS catalog are listed. Rare variants identified in the studies of Grosche et al.[9] and Mucha et al.[26] were added.

^2^ Allele frequency (AF) of the major allele in the European population of 1000 Genomes is reported.

^3^ Combined Annotation Dependent Depletion (CADD) score from model GRCh37-v1.4.

**Table 4:** Monogenic diseases with eczema-like symptoms.

Disease	Inheritance	MIM Number	Gene name (symbol)	Locus	Symptoms
Autoinflammation, immune dysregulation, and eosinophilia	Autosomal dominant	#618999	Janus kinase 1 (*JAK1*)	1p31.3	Characterized by severe eczema and chronic gastrointestinal inflammation. Patients tend to have asthma and food allergies as well as poor overall growth.
Ichthyosis vulgaris	Autosomal dominant	#146700	Filaggrin (*FLG*)	1q21.3	Palmar hyperlinearity, keratosis pilaris, and a fine scale that is most prominent over the lower abdomen, arms, and legs. Large proportion of patients with eczema, hay fever, or asthma.
Hyper-IgE recurrent infection syndrome-5	Autosomal recessive	#618944	Interleukin 6 receptor (*IL6R*)	1q21.3	Recurrent sinopulmonary and deep skin infections in early childhood. Additional features include eczema, impaired inflammatory responses during infection, increased serum IgE and IL-6.
Hyper-IgE recurrent infection syndrome-4A	Autosomal dominant	#619752	Interleukin 6 cytokine family signal transducer (*IL6ST*)	5q11.2	Recurrent sinopulmonary infections associated with increased serum IgE. Additional features include eczema, asthma, respiratory allergies, and eosinophilia.
Netherton syndrome	Autosomal recessive	#256500	Serine peptidase inhibitor Kazal type 5 (*SPINK5*)	5q32	Characterized by congenital erythroderma, a specific hair-shaft abnormality, and atopic manifestations with high IgE levels, such as eczema and hayfever.
Severe dermatitis, multiple allergies, and metabolic wasting syndrome (SAM)			Desmoplakin (*DSP*)	6p24.3	Severe eczema with multiple food allergies, persistent eosinophilia, and increased IgE levels.
Immunodeficiency 23	Autosomal recessive	#615816	Phosphoglucomutase 3 (*PGM3*)	6q14.1	Recurrent respiratory or cutaneous infections in early childhood, neutropenia, lymphopenia, eosinophilia, and increased serum IgE or IgA. Patients tend to develop atopic dermatitis, eczema, and autoinflammation.
Phosphoglucomutase 3 deficiency	Autosomal recessive		Phosphoglucomutase 3 (*PGM*3)	6q14.1	Characterized by eczema, skin and lung infections, elevated serum IgE as well as neurological and skeletal dysplasia.
Immunodeficiency 11B with atopic dermatitis	Autosomal dominant	#617638	Caspase recruitment domain family member 11 (*CARD11*)	7p22.2	Characterized by onset of moderate to severe atopic dermatitis in early childhood with defects in T-cell activation, increased IgE, and eosinophilia. Some patients may have recurrent infections.
Immunodeficiency 71 with inflammatory disease and congenital thrombocytopenia	Autosomal recessive	#617718	Actin related protein 2/3 complex subunit 1B (*ARPC1B*)	7q22.1	Onset of recurrent infections and inflammatory features, such as vasculitis and eczema in infancy or early childhood, thrombocytopenia, increased serum IgE, IgA, or IgM, leukocytosis, and increased eosinophils.
Hyper-IgE recurrent infections syndrome 2	Autosomal recessive	#243700	Dedicator of cytokinesis 8 (*DOCK8*)	9p24.3	Hyper-IgE, eosinophilia, and recurrent Staphylococcal infections, usually associated with eczema.
Immunodeficiency 41 with lymphoproliferation and autoimmunity	Autosomal recessive	#606367	Interleukin 2 receptor subunit alpha (*IL2RA*)	10p15.1	Recurrent viral, fungal, and bacterial infections in infancy, lymphadenopathy, and variable autoimmune features, such as autoimmune enteropathy and eczematous skin lesions.
Immunodeficiency 58	Autosomal recessive	#618131	Capping protein regulator and myosin 1 linker 2 (*CARMIL2*)	16q22.1	Early-onset skin lesions, including eczematous dermatitis, infectious abscesses, and warts, recurrent respiratory infections or allergies, and chronic persistent bacterial and viral infections. Some patients may have gastrointestinal involvement, including inflammatory bowel disease, EBV+ smooth muscle tumors, and esophagitis.
Hyper-IgE recurrent infections syndrome 1	Autosomal dominant	#147060	Signal transducer and activator of transcription 3 (*STAT3*)	17q21.2	Characterized by chronic eczema, recurrent Staphylococcal infections, increased serum IgE, and eosinophilia. Other immunologic abnormalities may include defective granulocyte chemotaxis, abnormalities in T-lymphocyte subgroups, impaired antibody production, and decreased production of or response to certain cytokines.
Hyper-IgE recurrent infections syndrome 3	Autosomal recessive	#618282	Zinc finger protein 341 (*ZNF341*)	20q11.22	Childhood onset of atopic dermatitis, skin infections particularly with Staphylococcus aureus, recurrent sinopulmonary infections, and increased serum IgE and IgG.
Immunodeficiency 63 with lymphoproliferation and autoimmunity	Autosomal recessive	#618495	Interleukin 2 receptor subunit beta (*IL2RB*)	22q11.3	Abnormal activation of certain immune signaling pathways, resulting in lymphoid proliferation, dermatitis, enteropathy, and hypergammaglobulinemia as well as features of immunodeficiency, such as recurrent infections and increased susceptibility to viral infections. Patients often develop eczema and food allergies.
Immunodeficiency 85 and autoimmunity	Autosomal dominant	#619510	Target of myb1 membrane trafficking protein 1 (*TOM1*)	22q12.3	Characterized by onset of atopic eczema and recurrent respiratory infections in the first decade of life, autoimmune enteropathy with vomiting, diarrhea, and poor overall growth.
Immunodysregulation, polyendocrinopathy, and enteropathy	X-linked recessive	#304790	Forkhead box P3 (*FOXP3*)	Xp11.23	Severe diarrhea due to enteropathy, type 1 diabetes mellitus, and dermatitis in infancy. Other features may include hypothyroidism, autoimmune hemolytic anemia, thrombocytopenia, lymphadenopathy, hepatitis, and nephritis. Most patients with eczema and increased serum IgE.
Wiskott-Aldrich syndrome	X-linked recessive	#301000	WASP actin nucleation promoting factor (*WAS*)	Xp11.23	Characterized by thrombocytopenia, eczema, and recurrent infections.

**Figure:**
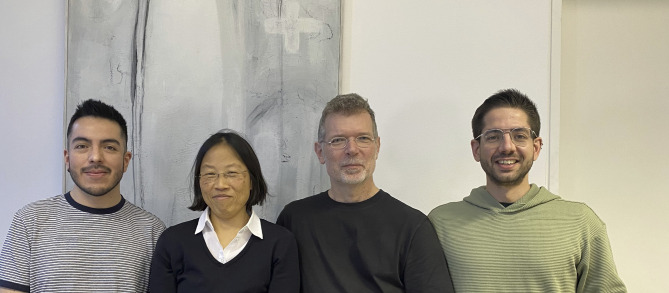
(von links nach rechts) Oscar Daniel Rosillo-Salazar, MSc, Young-Ae Lee, MD, Ingo Marenholz, PhD, Aleix Arnau-Soler, PhD
